# Genomic analysis of lncRNA and mRNA profiles in circulating exosomes of patients with rheumatic heart disease

**DOI:** 10.1242/bio.045633

**Published:** 2019-12-11

**Authors:** Yanli Luo, Lingjin Huang, Wanjun Luo, Shu Ye, Qinghua Hu

**Affiliations:** 1 Department of Anesthesiology, Xiangya Hospital, Central-South University, Changsha, Hunan Province, China 410008; 2 Department of Cardiovascular Surgery, Xiangya Hospital, Central-South University, Changsha, Hunan Province, China 410008; 3 Department of Dermatology, Hunan Children's Hospital, Changsha, Hunan Province, China 410007

**Keywords:** Microarray, Rheumatic heart disease, Exosome, LncRNA

## Abstract

Rheumatic heart disease (RHD) remains one of the most common cardiovascular conditions in developing countries. Accumulating evidence suggests that circulating exosomes and their cargoes, including mRNA and long noncoding RNA (lncRNA), play essential roles in many cardiovascular diseases. However, their specific roles in RHD remain unexplored. In the present study, we identified 231 lncRNAs and 179 mRNAs differentially expressed in the circulating exosomes harvested from RHD patients compared to healthy controls. We performed gene ontology (GO) and KEGG pathway analysis, and identified five pairs of lncRNAs and their flanking coding genes simultaneously dysregulated in the circulating exosomes. Collectively, we provide the first transcriptome analysis identifying differentially expressed lncRNAs and mRNAs in circulating exosomes of RHD patients, which may bring valuable insights for the discovery of potential biomarkers and therapeutic targets for RHD.

## Abstract (precis)

**Summary:** Differentially expressed lncRNA and mRNA profiles in circulating exosomes of patients with rheumatic heart disease compared to healthy control.

## INTRODUCTION

Rheumatic heart disease (RHD) is responsible for about 250,000 deaths per year worldwide, and particularly poses a great threat to people's health in developing countries (Marijon et al., 2012). RHD is generally characterized by progressive and permanent valvular lesions resulting from immune cross-reactions between streptococcal antigens and human proteins (Woldu and Bloomfield, 2016). However, the exact mechanisms leading to heart lesions remain unknown, partly due to lack of early diagnostic biomarkers (Guilherme and Kalil, 2010). Therefore, decisive biomarkers for RHD are yet to be discovered to facilitate early diagnosis.

A growing body of evidence has demonstrated that circulating exosomes, which are 30–120 nm membrane-bound vesicles secreted from tissue cells into the blood, can be employed as useful biomarkers for diagnosis and prognosis of many cardiovascular diseases (Bei et al., 2017a,b; Jansen and Li, 2017). Moreover, stem cell-derived exosomes or engineered exosomes can be potential tools to treat cardiovascular diseases (Huang et al., 2015; Mittal et al., 2018).

It is now well known that exosomes contain proteins, lipids, mRNA, miRNA and lncRNA (long noncoding RNA) that can be transferred from cell to cell either adjacently or remotely. One of the cargoes carried by exosomes are lncRNAs, which are >200-nucleotide long noncoding transcripts, classified into sense, antisense, intronic, intergenic, enhancer RNA and circular RNA. Accumulating evidence suggests that lncRNAs are essential for the regulation of tissue homeostasis and play important roles in cardiovascular diseases (Uchida and Dimmeler, 2015; Haemmig et al., 2017; Bär et al., 2016). For instance, in peripheral blood mononuclear cells, expression of myocardial infarction associated transcript (MIAT), which is involved with Wnt signaling pathway, was significantly reduced in ST-segment elevation myocardial infarction patients (Vausort et al., 2014). Similarly, long intergenic noncoding RNA predicting cardiac remodeling (LIPCAR) was shown to be downregulated after acute myocardial infarction but upregulated during late-stage heart failure, indicating that LIPCAR is associated with post-infarction cardiac remodeling and chronic heart failure (Kumarswamy et al., 2014). Certainly, our understanding of the roles of lncRNAs in cardiovascular diseases is still in its infancy and knowledge about the roles of lncRNAs in RHD is scarce.

In this study, we harvested serum exosomes from RHD patients and healthy controls, and investigated differential expression of lncRNA and mRNA between the two groups. A good number of differentially expressed lncRNAs and mRNAs were identified, of which some were verified by qPCR. We believe that these differentially expressed lncRNAs together with differentially expressed mRNAs of candidate genes can provide the basis for advancing our knowledge on the mechanisms of RHD development, and can serve as potential biomarkers and therapeutic targets for RHD.

## RESULTS

### Exosomes harvest and identification

**Fig. 1.**
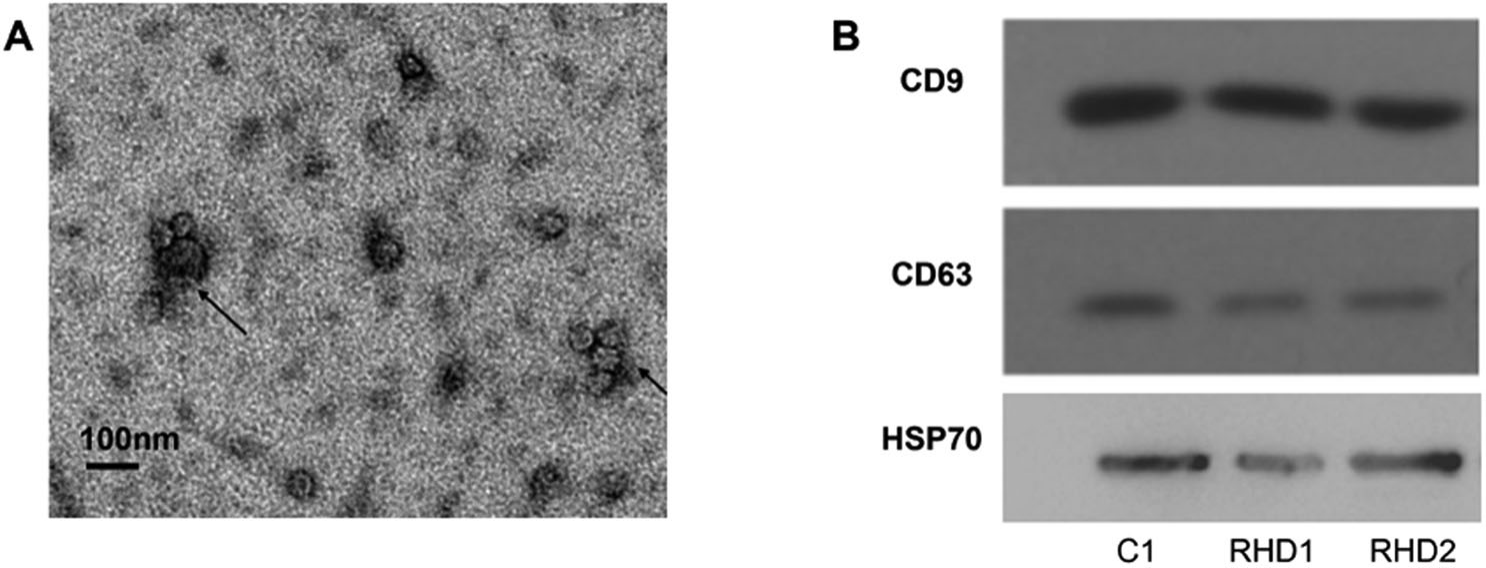
**Identification of exosomes.** (A) Results of scanning transmission electron microscope. Exosomes are indicated by arrows. (B) Results of western blotting. C1 from healthy control; RHD1,2 from rheumatic heart disease patients.

Under the scanning transmission electron microscope, we observed that the majority of the harvested exosomes presented as circular or oval cysts, whose diameters varied between 30–120 nm (Fig. 1A). The expression of CD9, CD63 and HSP70 by western blotting verified these structures to be exosomes (Fig. 1B). Taken together, the samples harvested in this study fit the characteristics of exosomes.

### Expression profile of lncRNAs and mRNAs in circulating exosomes

**Fig. 2.**
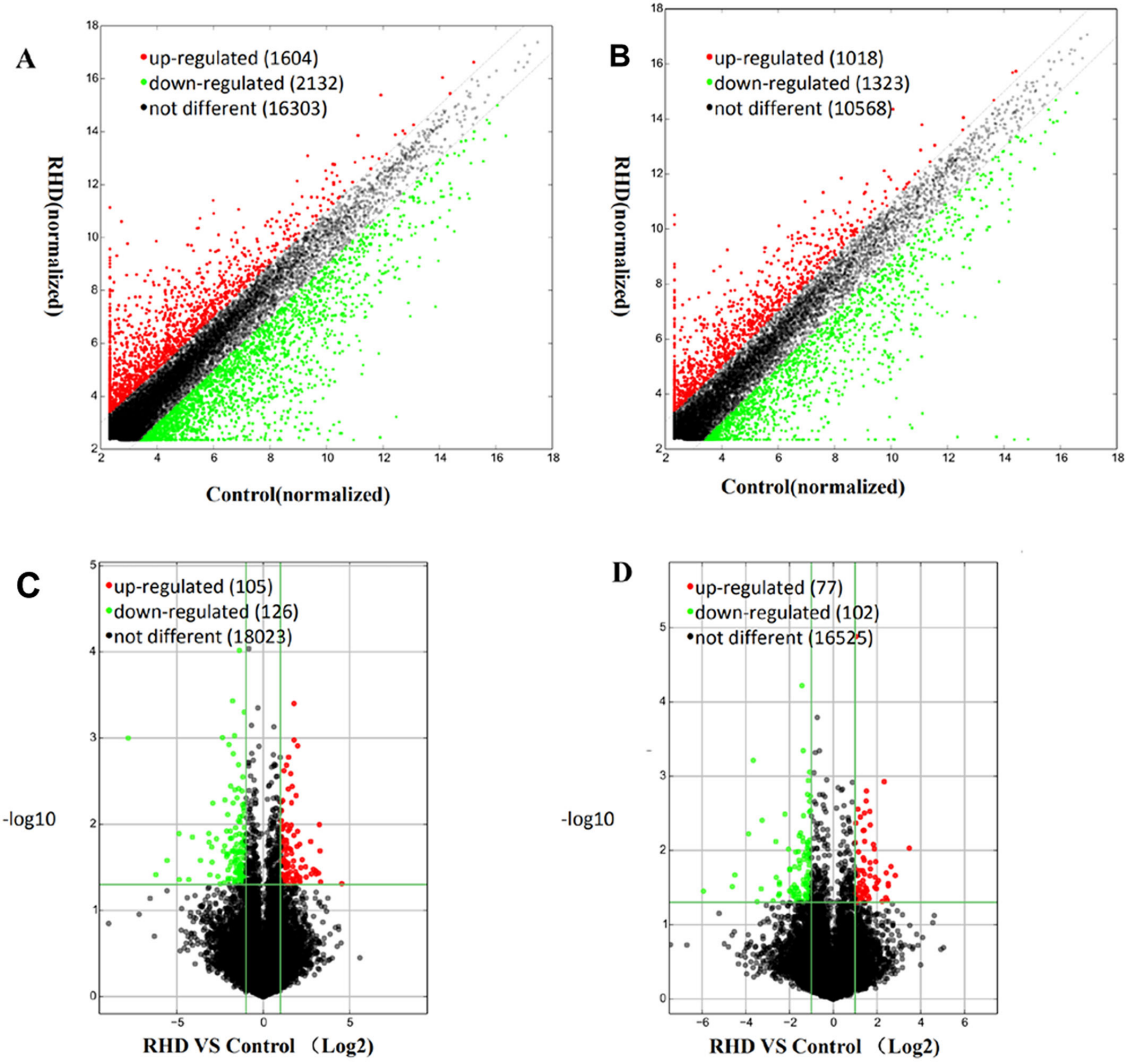
**Expression profiles of lncRNAs and mRNAs in circulating exosomes.** The scatterplots showed the profile of lncRNAs (A) and mRNAs (B). The volcano plot showed the profile of lncRNAs (C) and mRNAs (D). Red dots represent differentially upregulated expression and blue dots represent differentially downregulated expression (fold change≥2.0, *P*≤0.05, two-tailed *t*-test), while black dots indicate no difference.

To identify differentially expressed lncRNAs and mRNAs in the RHD group compared to the control group, we harvested total RNAs from circulating exosomes from RHD patients and healthy controls, and analyzed with LncRNA Microarray V4.0 platform. The expression profiles of lncRNAs (Fig. 2A) and mRNAs (Fig. 2B) that were upregulated, downregulated or unchanged between the two groups were displayed as scatterplots. Fig. 2C displays the expression profiles as volcano plot, which displays 105 significantly upregulated and 126 significantly downregulated lncRNAs in the RHD group. These differentially expressed lncRNAs covered almost the entire chromosome and varied greatly in length. In this study, intergenic lncRNA accounted for 45%, intronic sense accounted for 26%, intronic antisense accounted for 12%, natural antisense accounted for 15% and the remainder accounted for bi-directional transcripts from exons or introns. The top 20 upregulated or downregulated lncRNAs are listed in Table 1. As for differentially expressed mRNA, there were 77 significantly upregulated and 102 significantly downregulated in the RHD group compared to the healthy control (Fig. 2D), and the top 20 dysregulated mRNAs are presented in Table 2.

**Table 1.**
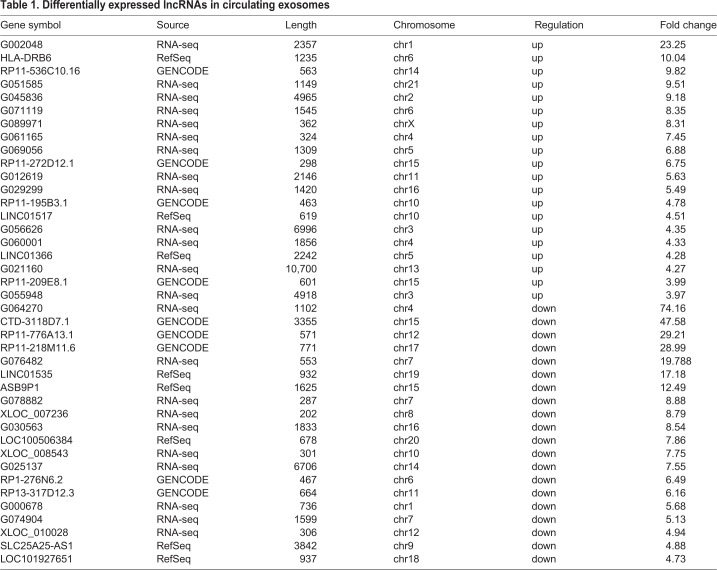
**Differentially expressed lncRNAs in circulating exosomes**

**Table 2.**
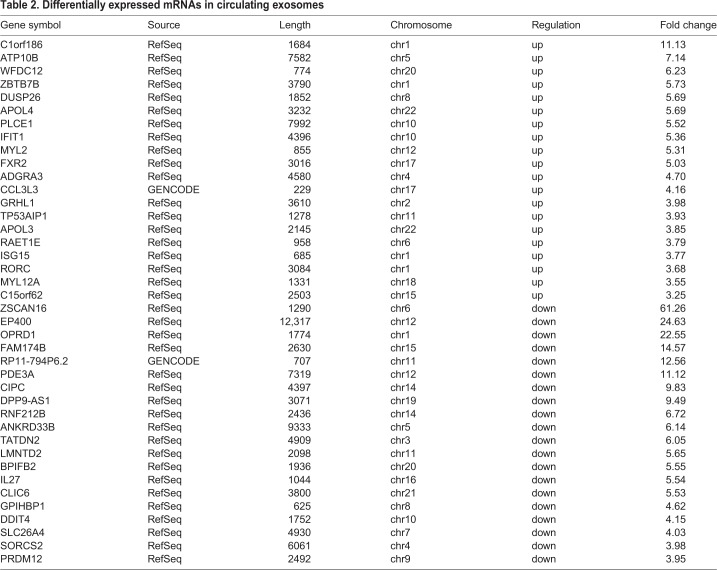
**Differentially expressed mRNAs in circulating exosomes**

The expression pattern of lncRNAs and mRNAs between the RHD group and the healthy control, as displayed by the heatmaps used for clustering analysis (Fig. S1), was very different. This result suggests that the presence of specific lncRNAs and mRNAs may be used to distinguish RHD patients from healthy controls.

### Validation of lncRNAs and mRNAs expression by qPCR

**Fig. 3.**
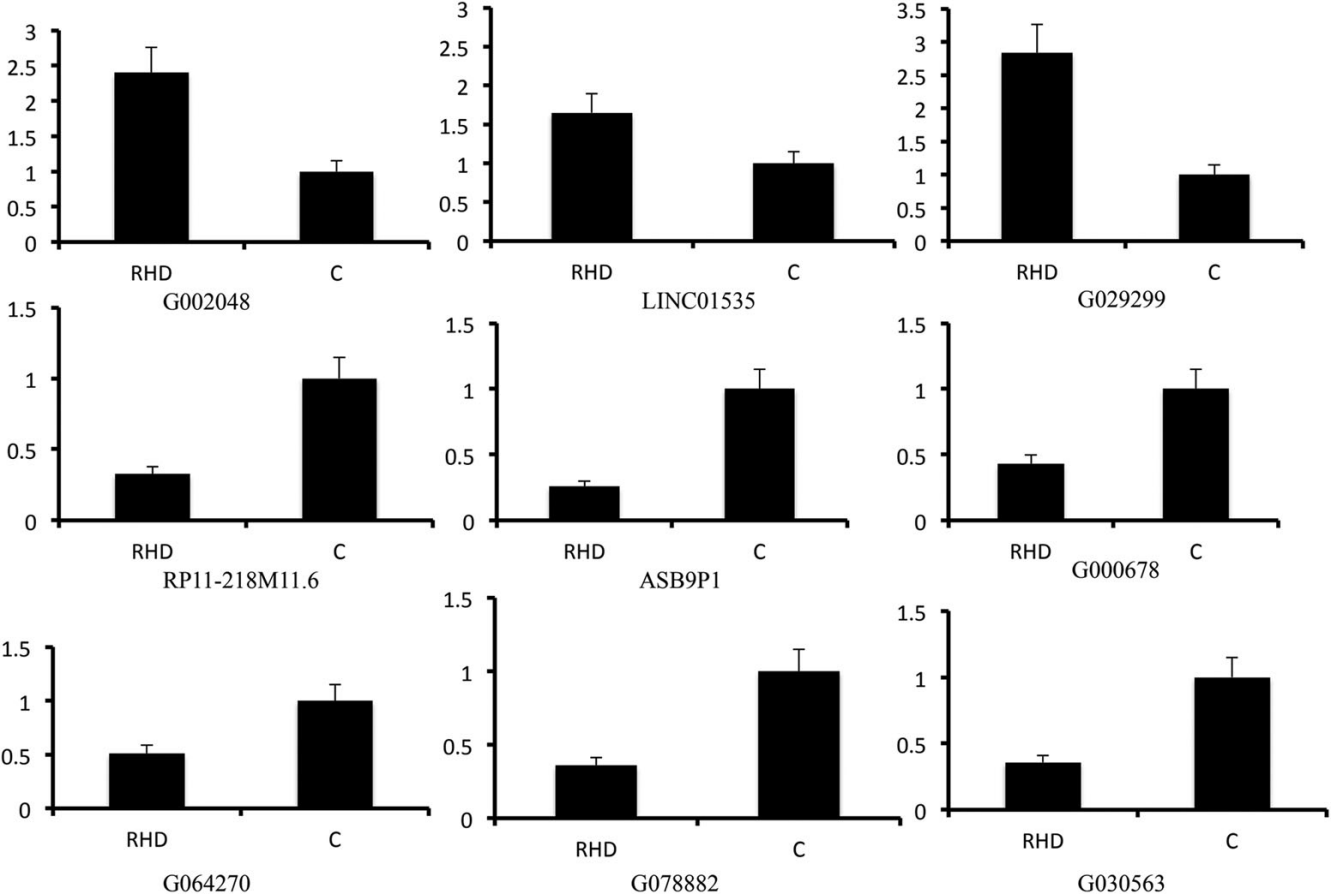
**Validation for selected lncRNAs by RT-PCR.** All *P*<0.05. RHD, rheumatic heart disease; C, control. (*n*=5 in each group, the significance was assessed with unpaired *t*-tests or Spearman analysis when appropriate).

Based on previous results of differentially expressed lncRNAs profile, we randomly chose nine lncRNAs, including three upregulated lncRNAs (G002048, LINC01535 and G029299) and six downregulated lncRNAs (RP11-218M11.6, ASB9P1, G000678, G064270, G078882 and G030563) to be validated by qPCR. As shown in Fig. 3, the qPCR results were consistent with the expression trend observed by the microarray analysis, although the fold changes were not exactly the same. Additionally, six differentially expressed mRNAs, including MYL12A, ZBTB7B, IFIT1, RPL22L1, CLIC6 and PIK3C3, were also validated by qPCR. Results from mRNA qPCR analysis were also in line with the results of the microarray analysis (Fig. S2).

### Gene ontology (GO) and pathway analysis

**Fig. 4.**
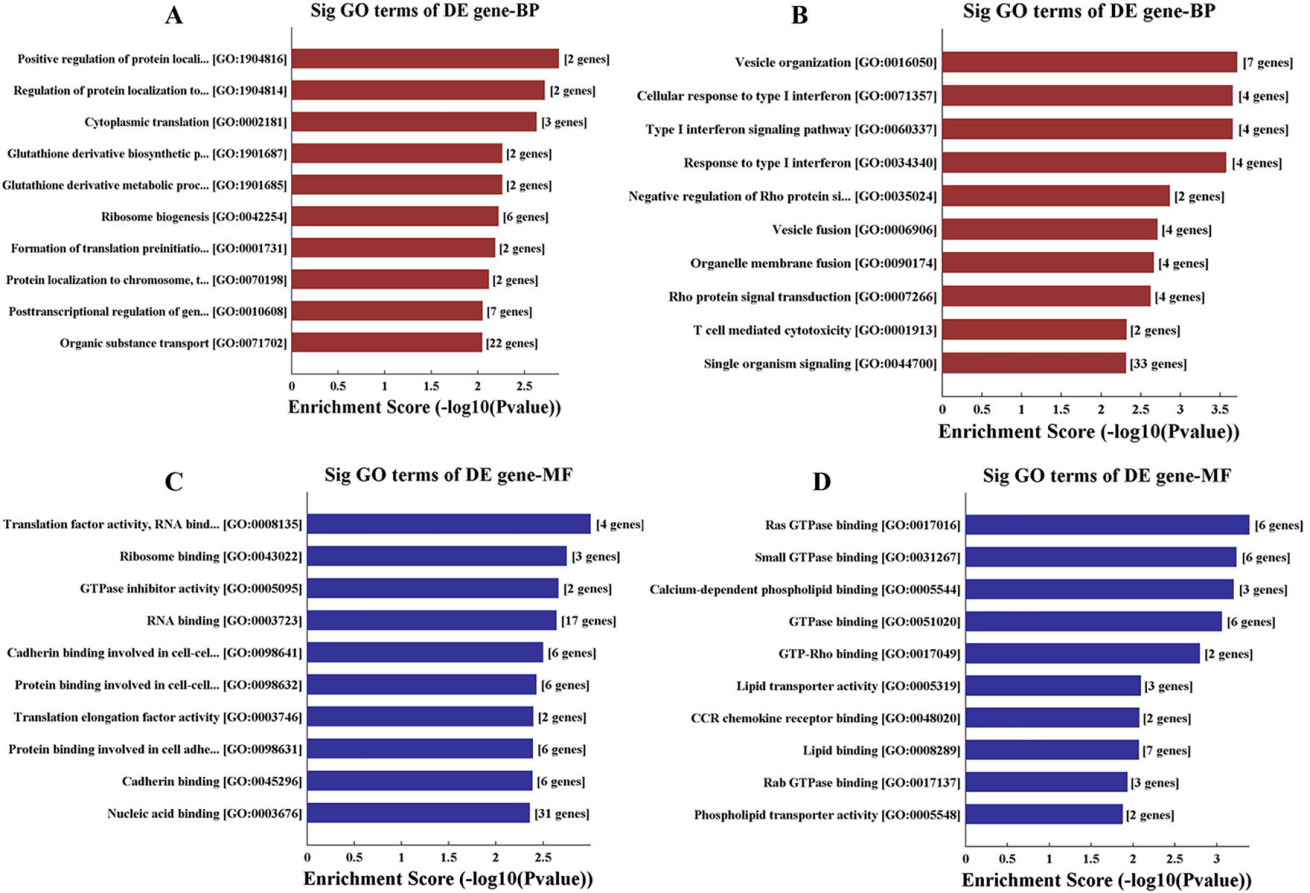
**Go analysis of the differentially expressed mRNA including downregulated mRNAs (A and B by biological process analysis) and upregulated mRNAs (C and D by molecular function analysis).**

The results from GO analysis of the differentially expressed mRNA including downregulated mRNAs (Fig. 4A,C) and upregulated mRNAs (Fig. 4B,D) are presented in Fig. 4. We found that the downregulated mRNAs were mostly involved in protein localization, ribosome biogenesis and metabolic process, while the upregulated mRNAs were mostly related to vesicle organization, type I interferon signaling pathway, T cell-mediated cytotoxicity and Rho protein signal transduction.

**Fig. 5.**
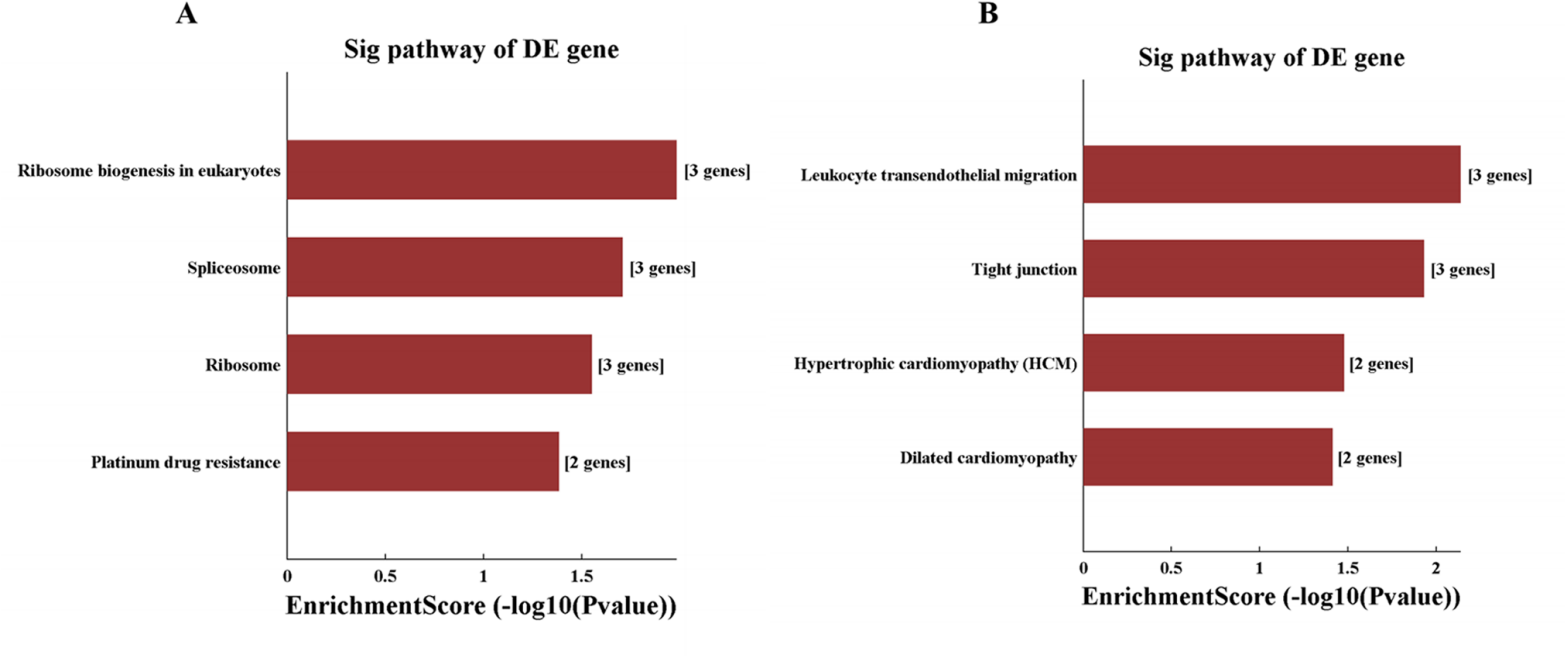
**Pathway analysis of the differentially expressed mRNA including downregulated mRNAs (A) and upregulated mRNAs (B).**

KEGG Pathway analysis (Fig. 5) revealed that the downregulated mRNAs mainly corresponded to the pathways associated with ribosome biogenesis, spliceosome, ribosome and platinum drug resistance whereas the upregulated mRNAs mainly corresponded to pathways associated with leukocyte transendothelial migration, tight junction, hypertrophic cardiomyopathy and dilated cardiomyopathy.

Additionally, we also performed GO analysis and pathway analysis of the associated nearby genes with differentially expressed lncRNAs, and found enrichment of pathways associated with magnesium homeostasis, ERBB signaling, Ras signaling, inflammation response, vesicle fusion and metabolism (Figs S3 and S4).

### Subgroup analysis

From the pools of differentially expressed lncRNAs and mRNAs in the circulating exosomes, we observed that five pairs of lncRNAs and their associated nearby genes were co-regulated, upregulating or downregulating simultaneously. As shown in Table 3, four pairs were downregulated and one pair was upregulated.

**Table 3.**
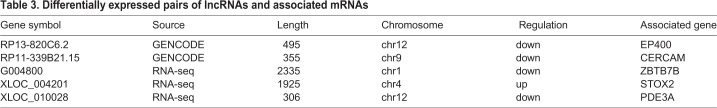
**Differentially expressed pairs of lncRNAs and associated mRNAs**

## DISCUSSION

Although RHD is not common in developed countries, it remains one of the leading causes of heart diseases in developing nations. In China, there are about 5 million RHD patients that bring remarkable economic and social burden to the nation (Cheng, 2009). Penicillin has been very useful to treat and prevent this disease if diagnosed early. However, early diagnosis of RHD has been difficult due to the failure to find sensitive and specific serum biomarkers. In the present study, we tried to open a new window for exploring potential biomarkers for this disease through genomic analysis of lncRNAs and mRNAs profile of circulating exosomes, which are associated with RHD.

Accumulating evidence demonstrates that circulating exosomes play essential roles, especially as intercellular communicators in the physiologic and pathophysiological conditions, including cardiovascular diseases. Many bioactive molecules such as protein, mRNA, miRNA and lncRNA are protected from being degraded in the serum when packaged in exosomes (Bei et al., 2017a). Our previous studies showed that in patients with RHD undergoing valve replacement surgery, remote ischemic conditioning could exert protective effects to multiple organs and could alter the expression profile of miRNAs in ischemic myocardium (Hu et al., 2016a,b). Vicencio and his colleagues speculated that the protective effect of remote ischemic conditioning mostly came from exosomes that served as intercellular communicators as the protective effects subsided greatly upon exosomes removal (Vicencio et al., 2015). However, how exosomes exert this function in RHD and how they change myocardial miRNAs expression remains elusive, and therefore, needs to be investigated.

We determined the lncRNA expression profile of serum-derived exosomes from RHD patients and healthy controls using LncRNA Microarray V4.0 platform. Using volcano plot filtering, we found that 231 lncRNAs were differentially expressed in the RHD group, including 105 upregulated and 126 downregulated lncRNAs. The majority of the differentially expressed lncRNAs do not have known functions, and thus, their bioactivities and functions need further investigation. Cluster analysis using heatmap revealed different expression patterns of these differentially expressed lncRNAs between the two groups, indicating that they may be potential biomarker candidates for early diagnosis of RHD. Furthermore, the rest of the lncRNAs transcripts derived from our comprehensive analysis of serum-derived-exosome lncRNAs transcripts from RHD patients will surely provide a promising resource for additional diagnostic purposes. For instance, LINC00969 found upregulated in RHD patients was shown to modulate the expression of microRNA195 (Boudreau et al., 2014). This finding may explain the results of our previous studies, where remote ischemic conditioning-induced downregulation of microRNA195 in myocardium greatly increased the production of circulating exosomes rich in LINC00969 (Hu et al., 2016b; Vicencio et al., 2015). Further experiments are certainly necessary to prove this hypothesis.

In the present study, we also examined the mRNA profile in the serum-derived exosomes and found that 179 mRNAs were differentially expressed in the RHD group, including 77 upregulated and 102 downregulated mRNAs. Same as the lncRNA profile, these differentially expressed mRNAs between RHD patient and healthy control presented as different clusters on heatmap, indicating that these mRNAs together with the differentially expressed lncRNAs can be potential biomarkers for RHD. Moreover, we used GO and KEGG pathway analysis for functional analysis of the differentially expressed mRNAs. GO analysis showed that the primary molecular functions of the differentially expressed mRNAs were protein localization, ribosome biogenesis, metabolic process, vesicle organization, type I interferon signaling pathway, T cell-mediated cytotoxicity and Rho protein signal transduction. Combining all aspects of our results demonstrated that RHD, in essence, is a metabolic and immune disease. Interestingly, KEGG pathway analysis revealed that the downregulated mRNAs mainly corresponded to pathways associated with non-specific biogenesis, whereas the upregulated mRNAs mainly corresponded to pathways associated with leukocyte trans-endothelial migration, tight junction, hypertrophic cardiomyopathy and dilated cardiomyopathy, which in turn are closely associated with the initiation and progression of RHD. Hence, our results also suggest that exosomes may play an important role in the development of RHD.

Furthermore, our subgroup analysis found that only five pairs of lncRNAs and their associated nearby genes were simultaneously dysregulated in circulating exosomes of RHD patients. We speculate that very few lncRNAs and their associated nearby genes are packaged together in the exosomes and that the majority of lncRNAs act as messengers to communicate with other cells rather than as regulators of target genes. In addition, such simultaneously dysregulated pairs may be of remarkable value for biomarker discovery for early diagnosis or progression of RHD disease. At present, the roles of all the five genes, including EP400, CERCAM, ZBTB7B, STOX2 and PDE3A, in RHD remain to be elucidated. Of note, ZBTB7B acts as a key regulator of lineage commitment of immature T-cell precursors and also functions as an important metabolic regulator of homeostatic adipose tissue remodeling (Kastner et al., 2010; Zhao et al., 2018). Hence, ZBTB7B is probably involved in the development of RHD disease and is worth further study.

Although our study was done with a low number of samples and was limited to exosomes collected from patients with RHD, and excluded other valvular heart diseases, our study provides the first transcriptome analysis identifying lncRNAs and mRNAs in seral exosomes in RHD patients. Our data may provide valuable insights to seek potential biomarkers and therapeutic targets for RHD and could contribute to develop novel strategies to prevent and treat RHD.

## MATERIALS AND METHODS

### Subject

We enrolled five patients (two male/three female, 52.6±4.8 years old) who were newly diagnosed with rheumatic mitral stenosis by typical echocardiographic findings in our institute between March to May 2017. Five age- and sex-matched healthy volunteers (two male/three female, 53.2±4.6 years old) were chosen as controls (See Table S1). All patients had New York Heart Association class II heart failure and had no medical history of any other diseases or previous drug usage. Through comprehensive physical and laboratory examination, we ruled out any patients with any diseases other than RHD. All patients later underwent mitral valve replacement surgery and the diagnosis of rheumatic heart disease was confirmed by open-heart surgery and the histological examination following the excision of valve tissue.

### Ethical approval

All procedures and protocols used in this study were approved by the Ethics Committee of Xiangya Hospital, Central-South University, China. All procedures performed in this study involving human participants were in accordance with the ethical standards of the institutional committee and with the 1964 Helsinki declaration and its later amendments. Prior to inclusion, written informed consent was obtained from each participant.

### Circulating exosomes harvest and identification

We withdrew 5 ml of blood from the peripheral vein of each patient and collected 2 ml plasma after centrifugation. We then harvested exosome using previously described ExoQuick Precipitation kit (SBI company, CA, USA) (Helwa et al., 2017). Briefly, 1.8 ml plasma was centrifuged at 3000× ***g*** for 15 min and the supernatant was collected. Then, the supernatant was filtered with a 0.22 μm filter. The ExoQuick Precipitation kit was used according to the manufacturer's instructions and the extracted portion was centrifuged at 1500× ***g*** for 5 min to collect the exosomes. The exosomes were re-suspended and stored at −80°C.

### Scanning transmission electron microscope

We used scanning transmission electron microscopy and western blotting to identify harvested exosomes. Exosome biomarkers such as CD9, CD63 and HSP70 were used as positive controls for western blot analysis.

Briefly, collected exosomes were incubated on the thin formvar/carbon film coated 200 mesh copper EM grids for 30 min and fixed with 3% glutaraldehyde in H_2_O for 5 min. After repeated washing, the grids were negatively stained with 4% uranyl acetate in 2% methyl cellulose in the dark and on ice for 10 min. Excess liquid was removed with a filter paper and images were acquired by TEM (Zeiss, Oberkochen, Germany) at 60 KV.

### Western blotting

After isolation, we lysed the exosomes in lithium dodecyl sulphate buffer (LDS buffer) and measured the protein concentration by BCA protein assay kit. Later, protein extracts were separated on SDS-PAGE, transferred to a PVDF membrane, and blocked with 5% milk in PBS and incubated overnight at 4°C with primary antibodies for CD9 (Abcam; EPR2949, 1:2000), CD63 (Santa Cruz Biotechnology; MX-49.129.5, 1:200), and HSP70 (Santa Cruz Biotechnology; sc-137210, 1:200). After washing, the membranes were incubated with the horseradish peroxidase-conjugated secondary antibody (Nanjing Jiancheng, 1:1000) for 1 h and washed again. Lastly, membranes were developed and images were collected on Bio-Rad Molecular Imager.

### RNA extraction and assessment

We extracted total RNA from exosomes by Trizol extraction kit (Invitrogen, Carlsbad, CA, USA) and purified total RNA using an RNeasy Mini Kit (Qiagen, Hilden, Germany) according to the manufacturer's instructions. RNA quantity and purity were measured by NanoDrop ND-1000 (Agilent, Santa Clara, CA, USA), and the absorbance ratios of OD260/280 were set between 1.8 and 2.1. The RNA integrity was assessed by standard denaturing agarose gel electrophoresis.

### RNA labeling and array hybridization

We performed RNA labeling and array hybridization according to the Agilent One-Color Microarray-Based Gene Expression Analysis protocol with minor modifications as described previously (You et al., 2015). Firstly, rRNA was removed from the total RNA using mRNA-ONLY™ Eukaryotic mRNA Isolation Kit (Epicentre, Chicago, IL, USA). Then, the purified mRNA was amplified and transcribed into fluorescent cRNA utilizing a random priming method (Arraystar Flash RNA Labeling Kit, Arraystar, Rockville, MD, USA). After purification, the quantity and purity of the labeled cRNAs were measured by NanoDrop ND-1000. 1 μg of each labeled cRNA was fragmented, heated and diluted. 50 μl of hybridization solution was assembled to the lncRNA expression microarray slide and incubated for 17 h at 65°C in an Agilent Hybridization Oven. The hybridized arrays were washed, fixed and scanned using the Agilent DNA Microarray Scanner (part number G2505C).

### Microarray analysis of lncRNA and mRNA expression

We used the Arraystar Human LncRNA Microarray V4.0 platform to assess the global profiling of human lncRNAs and protein-coding transcripts. The platform can detect about 40,173 lncRNAs and 20,730 coding transcripts from authoritative data sources, including ‘RefSeq’, ‘UCSC_knowngene’, ‘Genecode’, ‘lncRNAdb’, ‘lncRNA disease database’, ‘NRED’, ‘RNAdb’, ‘UCR’ and ‘RNA-seq’. Data were extracted and normalized using Agilent Feature Extraction Software. Volcano Plot filtering was used to identify differentially expressed lncRNAs and mRNAs that met the cut-off for statistical significance (*P*<0.05). The threshold was set to a fold change >2.0 (*P*<0.05), and was used to screen up or downregulated lncRNAs and mRNA.

### Quantitative real-time PCR

To evaluate the results from microarray analysis, we performed standard quantitative PCR (qPCR) on randomly selected lncRNA. Briefly, RNA samples were prepared as described above. Then, 1 μg of RNA from each sample was reverse transcribed into cDNA using SuperScriptTM III Reverse Transcriptase (Invitrogen) and dNTP mixture (HyTest Ltd. Turku, Finland). Real-time qPCR was performed in triplicate on ViiA7 Real-time PCR System (Applied Biosystems, Waltham, MA, USA). All primers (shown in Table S2) were designed by software Primer Premier 5.0 (PREMIER Biosoft International, Palo Alto, CA, USA). The expression level of each lncRNA or mRNA was normalized to the expression of β-actin and displayed as the fold change from β-actin.

### GO and pathway analysis

We performed GO analysis (http://www.geneontology.org/) and Kyoto Encyclopedia of Genes and Genomes (KEGG database) pathway analysis on differentially expressed mRNAs and associated nearby genes with differentially expressed lncRNA to identify enriched biological processes, cellular components and molecular functions.

### Statistical analysis

The statistical significance of the lncRNA and mRNA microarray datasets were analyzed by fold change and Student's unpaired *t*-tests and the false discover rate (FDR) was used to correct the *P*-value. We used a *P*-value cut-off of two-sided *P*<0.05 for statistical significance. Data from the quantitative real-time PCR experiments were analyzed with Student's unpaired *t*-tests or Spearman analysis when appropriate.

## Supplementary Material

Supplementary information
