# Supplementary material for: Genomic analysis of lncRNA and mRNA profiles in circulating exosomes of patients with rheumatic heart disease
Source: Biol Open. 2019 Dec 11;8(12):bio045633. doi: 10.1242/bio.045633 (PMC6918777; doi:10.1242/bio.045633)
Supplement: Supplementary information [file biolopen-8-045633-s1.pdf]

Table S1: Demographic data of RHD patients and healthy control.

	RHD group	healthy control	P value
Age (years)	52.6±4.8	53.2±4.6	>0.05
Weight (kg)	66.3±12.9	68.3±13.2	>0.05
Sex (female/male)	3/2	3/2	>0.05
Smoker (n)	0	0	>0.05
Drinker (n)	0	0	>0.05
History of diseases (n)	0	0	>0.05
Family diseases (n)	0	0	>0.05
NYHA class (n/class)	5/ II	5/I	<0.01
LVEF (%)	55.4±5.3	64.4±6.8	<0.01

RHD: rheumatic heart disease; NYHA: New York Heart Association class ; EF: left ventricular ejection fraction

Biology Open • Supplementary information

Table S2. Primers for selected lncRNAs.

Gene symbol	Primer sequence
G002048	F:5' GTGGCCGAGGACTTTGATTG3'
	R:5’ CCTGTAACAACGCATCTCATATT3’
LINC01535	F:5' AAGCATGTCTTTCAGAGCCC 3’
	R:5’ CGAGATCATCCAAATCCCTA 3’
G029299	F:5' ATGAGACACCTGGGAAGAGTG 3’
	R:5’ TGAGGGGACGTGAATTTGG 3’
RP11-218M11.6	F:5' TAAAAGAAGCAACGACACTCCC 3’
	R:5’ GCACAACACAAACCAAAGCC 3’
ASB9P1	F:5' TCATCTAATTCAGCCCTCACAG 3’
	R:5’ ATCCTTGCCCTGGGAAATT 3’
G000678	F:5' GGGTTCAGGTTGTTGTATTGTC 3’
	R:5’ GATTTGTTGTTCTGGTGGAGG 3’
G064270	F:5' CCCAGGATGTCCACAAAGAA 3’
	R:5’ GCTCATACACCGTGAAGGCT 3’
G078882	F:5' TAACTTGCCTCAGTCTCTCCCT 3’
	R:5’ ACTCACAGCAACTTCAGTCCTT 3’
G030563	F:5' CCAAATGTCCCCAGAGTGAC 3’
	R:5’ TGAGGTGTTCGCTCGCTTT 3’
β-actin（H）	F:5' GTGGCCGAGGACTTTGATTG3'
	R:5’ CCTGTAACAACGCATCTCATATT3’

Biology Open • Supplementary information

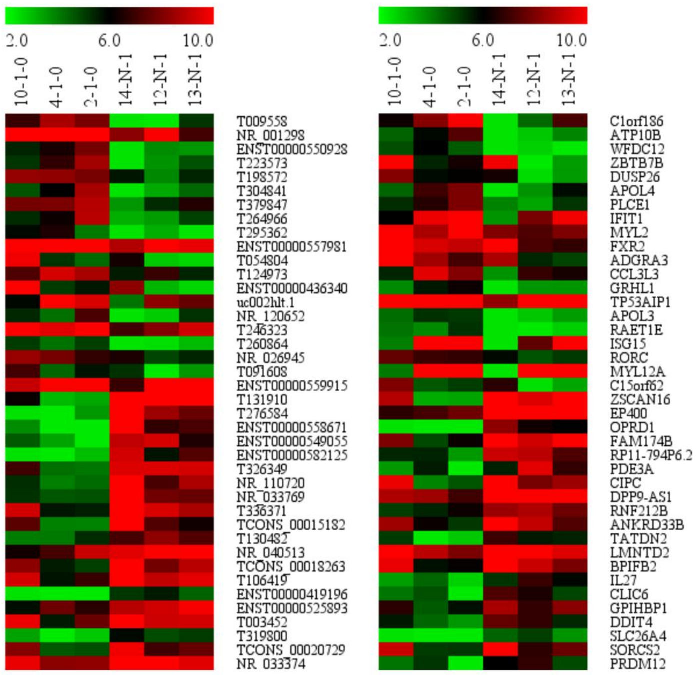

Figure S1. Heatmaps of differentially expressed lncRNAs (left) and mRNAs (right). 10-1-0, 4-1-0 and 2-1-0 were sample number from RHD group while 14-N-1, 12-N-1 and 13-N-1 from control group.

Biology Open • Supplementary information

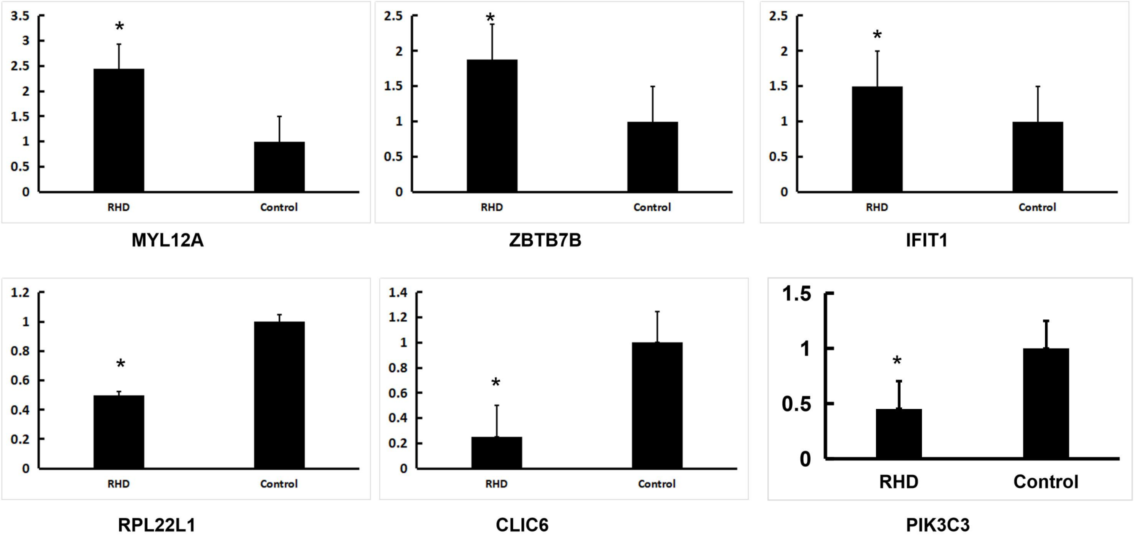

Figure S2. Validation for selected mRNAs by RT-PCR. * $p < 0.05$. RHD rheumatic heart disease; C healthy control.

Biology Open • Supplementary information

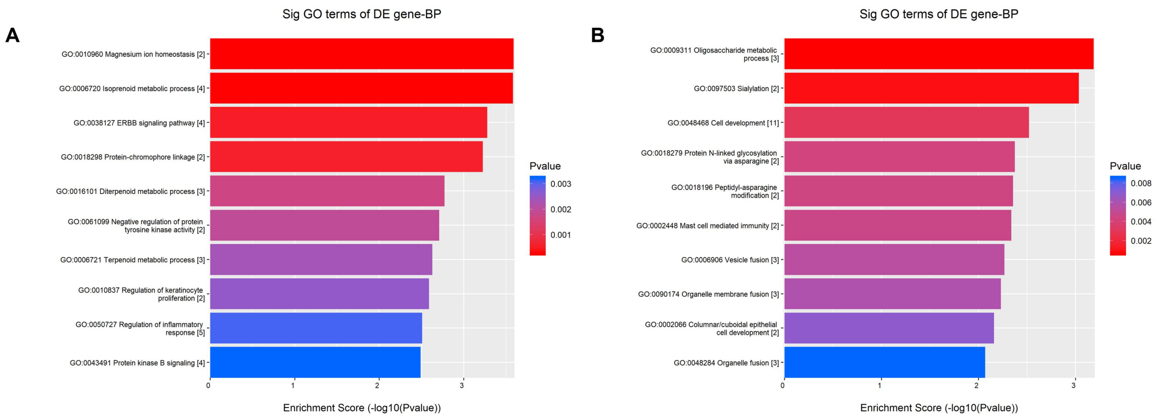

Figure S3. Go analysis of the associated nearby genes with differentially expressed lncRNA downregulated (A） or upregulated （B）.

Biology Open • Supplementary information

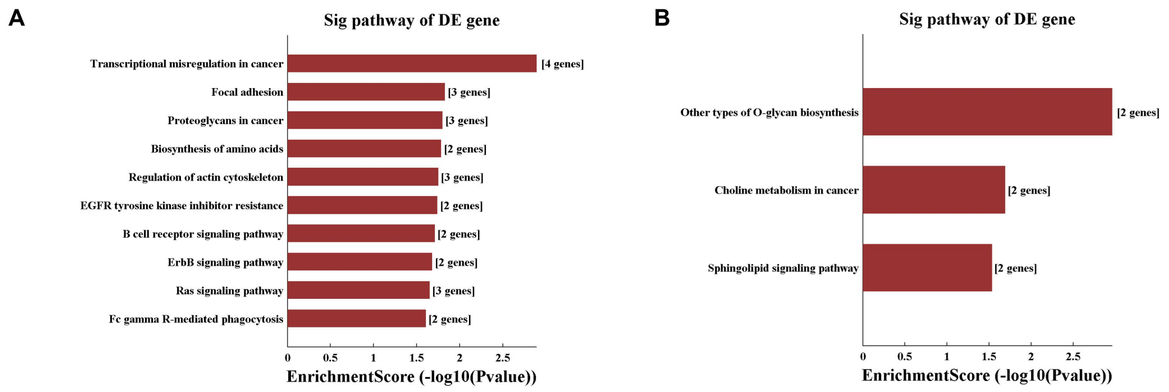

Figure S4. Pathway analysis of the associated nearby genes with differentially expressed lncRNA downregulated (A) or upregulated (B).

Biology Open • Supplementary information
